# Anti-Typhoid Inoculation

**Published:** 1900-06-30

**Authors:** 


### ANTI-TYPHOID INOCULATION.

We do not exaggerate when we say that the whole nation has been greatly moved by the extraordinary epidemic of typhoid fever which has lately held within its grip so large a proportion of our army in South Africa; and there is no doubt that much questioning is going on as to the utility of the anti-typhoid inoculation which has been so extensively employed for its prevention. Until we have before us the complete records which will tell whether there has been any difference in the incidence of the disease on the inoculated and the non-inoculated it is impossible to speak with certainty as to the degree of protection, if any, afforded by the process, but cases of fever among the inoculated have already been reported in sufficient number to show—what, indeed, was to be expected—that no complete and reliable prophylactic has yet been discovered. It has to be recognised, however, that the proceeding has throughout been of the nature of an experiment, and that, although much was to be hoped from it, nothing could be promised; and under the circumstances, and in view of the great interest given to the whole subject by the recent events in South Africa, it may be well to recall what are the data on which the experiment is based, and what are the limitations to its possible usefulness.

Put in general terms it may be stated that the blood is so altered when an animal suffers from an infectious disease that for some time—may be for life—it remains resistent to the same infection. This is an invisible change, the nature of which we can but guess at, but at the same time a change takes place which is capable of demonstration, for it is found that the serum of such an animal has obtained the property of producing a distinct reaction when added to a fluid culture of the same infective micro-organism as those by which the disease was produced. Thus we have the well-know Widal's test for typhoid fever, the reaction consisting of a "clumping" or "agglutination" of the microbes followed by their rapid "sedimentation," so that the milky fluid quickly becomes clear. By this test we can tell with a considerable degree of positiveness whether a given patient has suffered lately from typhoid fever.

The general principle of all vaccination is that it aims at producing in the tissues and blood that same protective change which otherwise only results from going through the complete disease, and one way of attaining this end is to inoculate with a sterilised culture of the microbes by which the disease is produced. This is the method employed when inoculating against typhoid. Now we come back to Widal's test. It is found that in persons so "vaccinated" or inoculated with sterilised cultures of typhoid organisms, the blood reacts just as if they had had typhoid fever. In other words, we are able by such an inoculation to produce the same condition of blood, so far as this test is concerned, as is produced by the full disease. But is the patient therefore immune? Is his blood in such a condition that it will kill off any typhoid bacilli which may obtain access to it? To this we may certainly answer "No." This is capable of complete experimental proof, so that we must not pretend to speak of anything like absolute immunity.

May we, however, infer that, short of immunity, an individual so inoculated that his blood reacts properly to Widal's test is more or less protected against the disease? The answer to this, so far as it goes, is partly derived from analogy, partly from statistics. It seems that guinea-pigs, on whom this reaction has been conferred by such inoculation, are extremely resistent to infection by the particular micro-organism with respect to which their blood possesses this agglutinating and sedimenting power, and the same has been found with other animals. Even in man experiments have been performed, showing that when well marked reaction has been produced by repeated anti-typhoid inoculation, virulent cultures containing living typhoid bacilli have failed to set up typhoid fever. In regard to statistics we need only refer to comparatively recent articles in which the results obtained from several series of inoculations undertaken in India are described. The number of troops in regard to whom these observations were made amounted to 11,295, of whom 2,135 submitted to inoculation, and the outcome was that during the period through which the results were watched the percentage of cases of typhoid among the inoculated was 0·95 against 2·5 among the uninoculated.

Take it altogether, then, the position is, that analogy, statistics, and a small amount of direct proving, all tend to suggest that some degree of protection may be gained by anti-typhoid inoculation. That is as far as we can get. But when we are asked what degree? and how long does it last? we are fain to confess that we do not know; and here we come upon what to many will seem the weak spot in the whole affair, namely, that while we cannot reasonably expect a protection produced by vaccination to be any greater than that which results from going through the disease itself, we do not know with anything like certainty how far going through a complete attack of typhoid fever does produce protection against a repetition of the disease. Of course some degree of immunity is produced, but we are afraid that no one can say how long it will last, and certainly no one can maintain that if a man's blood still "reacts" he is protected. One must admit then that there is nothing surprising in the occurrence of even a considerable number of cases of typhoid fever among the inoculated troops in South Africa. The event is much to be regretted, but it is far too early to say, as some seem ready to do, that inoculation has therefore been proved a failure. It has not failed because it has not promised. The test will come with the statistics, and cannot come till then; and till then all individual experience, however interesting, is beside the mark.

